# Integration of artificial intelligence performance prediction and learning analytics to improve student learning in online engineering course

**DOI:** 10.1186/s41239-022-00372-4

**Published:** 2023-01-17

**Authors:** Fan Ouyang, Mian Wu, Luyi Zheng, Liyin Zhang, Pengcheng Jiao

**Affiliations:** 1 College of Education, Zhejiang University, Hangzhou, 310058 Zhejiang China; 2 Institute of Port, Coastal and Offshore Engineering, Ocean College, Zhejiang University, Zhoushan, 316021 Zhejiang China

**Keywords:** Artificial intelligence in education (AIEd), Academic performance prediction, AI prediction models, Collaborative learning, Online higher education

## Abstract

As a cutting-edge field of artificial intelligence in education (AIEd) that depends on advanced computing technologies, AI performance prediction model is widely used to identify at-risk students that tend to fail, establish student-centered learning pathways, and optimize instructional design and development. A majority of the existing AI prediction models focus on the development and optimization of the accuracy of AI algorithms rather than applying AI models to provide student with in-time and continuous feedback and improve the students’ learning quality. To fill this gap, this research integrated an AI performance prediction model with learning analytics approaches with a goal to improve student learning effects in a collaborative learning context. Quasi-experimental research was conducted in an online engineering course to examine the differences of students’ collaborative learning effect with and without the support of the integrated approach. Results showed that the integrated approach increased student engagement, improved collaborative learning performances, and strengthen student satisfactions about learning. This research made contributions to proposing an integrated approach of AI models and learning analytics (LA) feedback and providing paradigmatic implications for future development of AI-driven learning analytics.

## Highlights

- Integrated approach was used to combine AI with learning analytics (LA) feedback
- Quasi-experiment research was conducted to investigate student learning effects
- Integrated approach to foster student engagement, performances and satisfactions
- Paradigmatic implication was proposed for develop AI-driven learning analytics
- Closed loop was established for both AI model development and educational application.

## Introduction

Next-generation educational technologies have led to the extensive applications of computers and information and related computing technologies in education, such as artificial intelligence in education (AIEd) (Chassignal et al., 2018; Chen et al., 2020; Zawacki-Richter et al., 2019). AIEd takes advantage of immense data processing and analytics to enable human-like cognition and functionalities, which has become a field of scientific research to improve online education or blended learning. Particularly, AIEd has significantly promoted the emergence of new functionalities in education such as learning performance prediction (Jiao et al., 2022; Liao, et al., 2022), learning path recommendation (Nabizadeh et al., 2020), teaching strategy optimization (Taheri et al., 2021; Toyoda et al., 2022), etc. AI-enabled academic performance prediction is one of the cutting-edge applications in AIEd, which assists in identifying the students that tend to fail, establishing student-centered learning pathways to improve learning effectiveness, and optimizing instructional design and development (Mozer et al., 2019; Nabizadeh et al., 2020; Taheri et al., 2021).

The AI performance prediction models can be categorized with respect to two perspectives in a closed loop, including (1) *From the AI model perspective* that mainly focuses on improving the accuracy of AI prediction models: development and validation of AI models to accurately predict students’ learning performance, and (2) *From the educational application perspective* that mainly focuses on the application and effect of AI prediction models: application of AI prediction models to effectively help instructors and students improve the quality of teaching and learning. Successful AI-enabled academic performance prediction models developed from these two perspectives should form a loop of AI model development and optimization as well as educational application and validation through empirical research (Wu et al., 2022; Xie et al., 2019; Yang et al., 2021). However, a majority of the existing studies on AI prediction models have mostly focused on the development and optimization of AI models, i.e., using multiple algorithms to develop models with higher prediction accuracy (e.g., Jiao et al., 2022). Furthermore, although there is a research trend to carrying out on the educational application of AI prediction models in practice, there is a lack of applying AI prediction model that integrates in-time feedback and offers appropriate feedback to the instructors and students in order to improve students’ learning quality. The recent research trend of the integration of AI and learning analytics (LA) has potential to address this issue (Darvishi et al., 2022; de Carvalho & Zárate, 2020; Starcic, 2019).

To address the research and practice gap, this research proposed an integrate approach by offering student performance generated by an AI performance prediction model and in-time LA feedback with a goal to foster students’ learning effects. This research further conducted quasi-experimental research in an online engineering course to compare the difference of student learning with and without the support of the integrated approach. Based on the results, this research proposed implications for future integration of AI model and learning analytics and made efforts to establish the paradigms for completing the closed loop of AI development and educational application.

## Literature review

### Accuracy of AI prediction models: From the AI model perspective

Online higher education has attracted extensive attention in the COVID-19 period with a goal to improving the quality of personalization, monitoring and evaluation in learning (Hwang et al., 2020). AI performance prediction model has been used as a promising method in online higher education to accurately predict and monitor students’ learning performance using student learning data and AI algorithms (Aydogdu, 2021; Sandoval et al., 2018; Tomasevic et al., 2020). The existing AI performance prediction models have been developed from the AI model perspective: with the objective of predicting the learning performance that students are likely to achieve given all the input information (Cen et al., 2016). A majority of relevant research is found on the choice of AI algorithms, examination of the accuracy of AI models, as well as validations of the AI models for performance prediction (Lau et a., 2019).

Studies have been conducted to apply advanced AI algorithms (including Machine Learning ML, evolutionary computation EC) and improve the accuracy of AI prediction models, such as ML algorithms—Bayesian network, decision trees, support vector machines, artificial neural networks, deep learning, and EC algorithms – Genetic programming, etc. (Fok et al., 2018; Fung et al., 2004; Jiao et al., 2022; Sharabiani et al., 2014). Naïve Bayes classifier algorithm was used to develop a predictive modelling method to identify at-risk students in a class that used standards-based grading (Marbouti et al., 2016). Later, Bayesian updating approach was used to optimize the predictive modelling method with higher model transferability over time within the same class and across different classes (Xing et al., 2021). Deep artificial neural network (ANN) was deployed to predict at-risk students with early intervention using the unique handcrafted features and data extracted from the virtual learning environments (Waheed et al., 2020). Among all the AI algorithms for learning performance prediction in online education, machine learning (ML) has been considered as one of the most applicable series of algorithms in recent research (Tomasevic et al., 2020). For instance, Jiao et al. (2022) proposed prediction criteria to characterize and convert the learning data in an online engineering course and developed an evolutionary computation technique—genetic programming—to explore the best prediction model for the student academic performance. In summary, the choice of AI algorithms in developing AI models is one of the major considerations.

Another important consideration of improving the accuracy of AI prediction is to establish the specific criteria to define the variables for characterizing the learning processes. In existing research, available student information data (e.g., age, gender, religion, place of living, job, grades, etc.) are typically considered in the prediction models (Asif et al., 2017), rather than using the data that reflect the specific learning process (Suthers & Verbert, 2013). For example, the most frequently used input data in the prediction models include students’ prior performance, engagement level, and demographic information (Lam et al., 1999; Nicholls et al., 2010; Tomasevic et al., 2020). Recently, there are emerging studies that focus on using online learning behavior data from the process-oriented perspective to improve accuracy of prediction model, rather than merely using student information data (e.g., demographics) or performance data (e.g., final grades) (Bernacki et al., 2020). Particularly, because collaborative learning emphasizes the importance of peer interaction and communication, the process-oriented attributes can better reflect the quality of collaborative learning than the individual-level information alone (Damşa, 2014; Dillenbourg, 1999). For example, specific variable selection criteria of collaborative learning process data were reported on online courses, including student participation frequency, procedural performance, discussion depth and the results demonstrated the accuracy and efficiency of the developed prediction model with the integration of procedural and summative data (Jiao et al., 2022). In summary, a majority of the existing studies on AI prediction models have focused on the AI model perspective, i.e., using multiple algorithms to develop models with higher prediction accuracy.

### Effect of AI performance prediction models: From the educational application perspective

Online higher education has been improved by different types of AI prediction models such as early warning systems, recommender systems, and tutoring and learner models (Sandoval et al., 2018). As an important component of AI prediction models, providing feedback to the instructors and students have become a critical strand in recent research (Bravo-Agapito et al., 2021). For example, an early warning system was developed based on the prediction results of an adaptive predictive model to provide early feedback and intervention for at-risk students (Baneres et al., 2019). A dropout prediction model was developed for at-risk students in MOOCs and the model was optimized by providing personalized interventions that took into account the situations and preferences of individual students (Xing & Du, 2019). Overall, from the educational application perspective, appropriate feedback and intervention based on AI prediction results has significant influences on improving the effect of AI prediction models in teaching and learning processes.

However, existing research neither considers the dynamic influence of students’ learning progress on their performance, nor do they provide in-time feedback to the instructor and students regarding the procedural learning performance. Some existing studies have been conducted to investigate the influence with respect to the subject specific attributes and general attributes (Yang & Li, 2018). On the one hand, the subject specific attributes mainly evaluate the progress of students on understanding of certain subject learning materials; under this circumstance, AI prediction models were used to estimate the level of students’ understanding against the difficulties in the learning materials (Mitrovic, 2001). The AI models are used to assist in identifying the reasons why students give up addressing a particular problem and the capability of students to find the method of solving the problem (Guzman et al., 2007). On the other hand, general attributes are also considered, which refer to the non-subject related attributes such as assessment of feasibility, creativity, etc. (Yang & Tsai, 2010). The AI models are able to provide the prediction feedback on certain aspects of attributes, but are rarely to provide a global view showing how improvement can be made after taking different subjects or learning activities in specific educational applications (Yang & Li, 2018). Therefore, the existing AI prediction models are less effective in fostering students’ learning processes and optimizing the instructor’s pedagogical strategy with the prediction results. To improve teaching and learning effects, further research of AI prediction models should provide and integrate the in-time feedback generated by the models and offer optimization suggestions to close the loop of AI model application and educational intervention in the actual teaching and learning process.

### An integration of AI and LA approaches: Moving from the AI model to the educational application perspective

Traditional teaching and learning follows learning taxonomies, which are more domain-specific and outcome-related; but in the current AIEd age, the appropriate integration of AI and LA can support personalized, adaptive, process-oriented instruction and learning (Luckin & Cukurova, 2019). Particularly, the integration of AI and LA has potential to provide both quantitative performance generated by AI model and qualitative feedback from the instructor’s or researcher’s perspectives, which can further improve student learning process and performance. For example, Darvishi et al. (2022) incorporated AI and LA to build a trustworthy peer assessment system and this research showed that this AI-assisted and analytical approaches integrated AI model to improve the accuracy of the assigned task for the instructors. Students, supported with the integrated AI and LA techniques, successfully wrote lengthier and more helpful feedback comments to peers, which improved their learning effects and final performances. Sullivan and Keith (2019) proposed and applied an interdisciplinary method that combines natural language processing techniques with qualitative coding approaches to support analysis of student collaborative learning. This research argued that machine learning techniques assisted with computational linguists’ support better analyzed conversational dialogues that are ill-structured, indexical and referential. Chango et al. (2021) reported that attribute selection and classification-ensembled AI algorithms improved the prediction effects of students’ final performance with multimodal learning data source and analytics from the intelligent tutoring systems. Therefore, an integration of AI and LA can help move from the AI model perspective to the educational application perspective. Particularly, AI can automatically capture and analyze the learning process and the learner’s psychological states, and LA can provide relevant feedback and suggestions from the educators or practitioners concerning the cognitive process, social interaction, and affectional or metacognitive states (Starcic, 2019). More importantly, relevant literature indicates that the integrated AI and LA techniques have potential to eventually improve students’ learning performances (e.g.,Chango et al., 2021; Darvishi et al., 2022).

Given the current situation of AI prediction models related to the data identifications, data analytics, and educational applications, future trend of AI prediction models should move from the optimization of AI models and algorithms to authentic application and intervention of AI models. Most existing research has focused on the development and optimization of AI models and related algorithms; however, few research has provided AI-driven learning analytics feedback based on results generated by the AI models and few research has examined the actual effects of AI models in educational practice. The existing AI prediction models experience challenges in the educational applications due to the following two dilemmas. First, there is a lack of applying AI prediction models to support the teaching and learning procedures; and second, difficulties are found in optimizing actual teaching and learning quality based on the in-time feedback generated by AI models. The abovementioned integrated approach can combine the data generated from the AI performance prediction model with the support of learning analytics feedback or guidance to enable instructors and students to gain insights into the learning processes (de Carvalho & Zárate, 2020; Luckin & Cukurova, 2019; Starcic, 2019). This research explore the potential of using AI performance prediction model and learning analytics to address some of the common concerns discussed above as a potential approach to close the loop of AI prediction models and educational application, integrate AI and LA approaches to support learning, and investigate the empirical effect of the integrated approach on learning.

## Research methodology

### Research purpose and question

The research purposes were to apply an AI-enabled academic performance prediction model designed by the research team in an online engineering course, and to visualize the results generated by the prediction model to the course instructor and students in order to improve teaching and learning quality. Furthermore, this research empirically examined the effects of this AI prediction model on student learning with quasi-experimental research. The research questions were:

*RQ1. To what extent did two conditions differentiate in terms of students’ engagement?*

*RQ2. To what extent did two conditions differentiate in terms of students’ procedural and final performances?*

*RQ3. To what extent did two conditions differentiate in terms of students’ perceptions about their learning experiences?*

### Research context and participants

The research context was an 8-week, online graduate-level course titled “*Smart Marine Structure*” offered in the summer semester in 2022 at a top-tier university in China. This course centered on the application of artificial intelligence in ocean engineering with particular focuses on the advanced technologies in mechanical metamaterials, structural health monitoring systems, and artificial intelligence in engineering. There were two classes per week over an eight-week period, and each class lasted for 90 min. The course was a completely online course and the online teaching and learning platform was DingTalk. Participants were 62 graduate students (43 master students and 19 doctoral students), majoring in ocean engineering at Ocean College from the university. The students were assigned into 15 groups (four to five students per group), mixed with master and doctoral students in each group.

### Instructional procedure

**Fig. 1:**
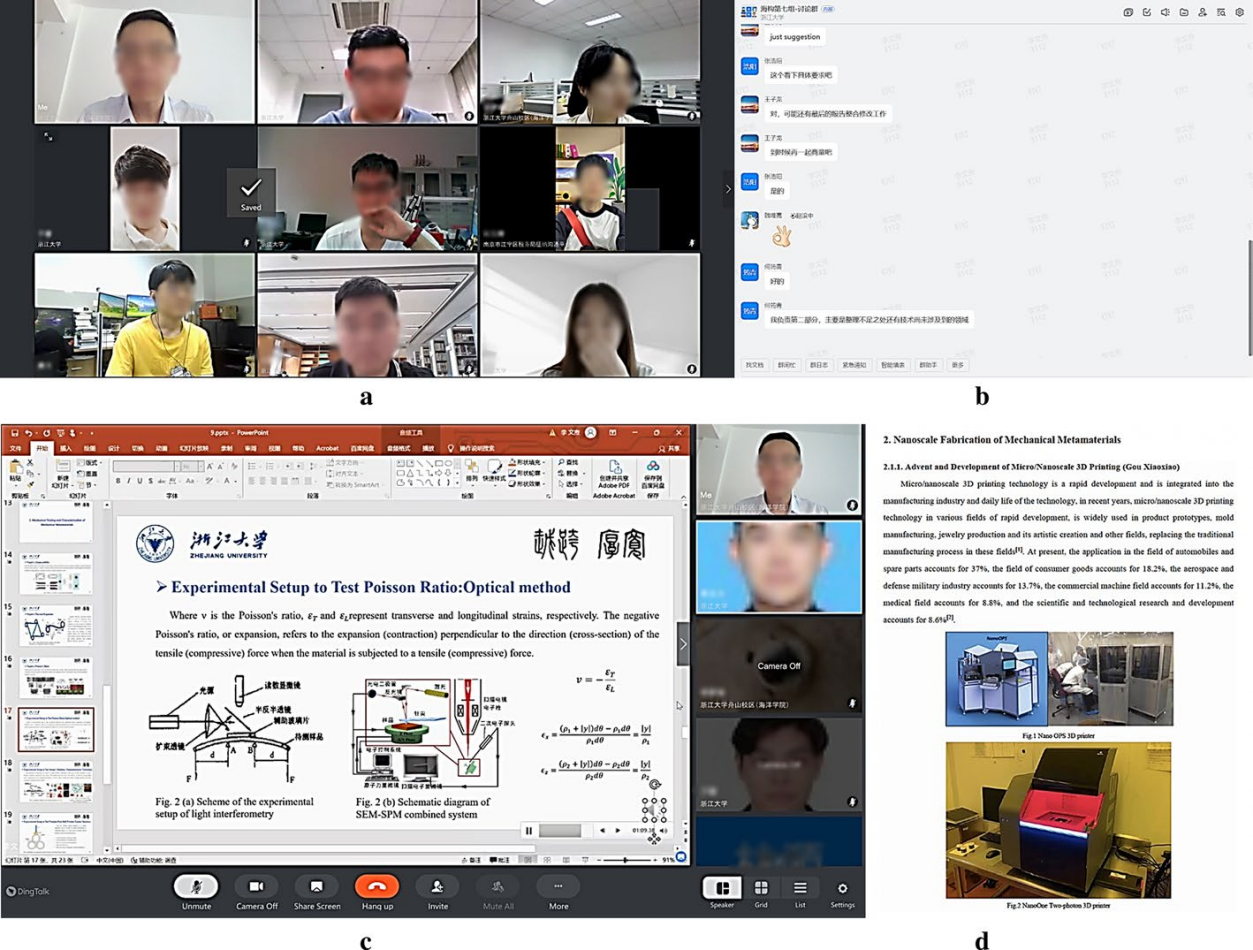
Screenshots of instruction and learning procedures

Except the orientation week (Week 1) and the final presentation week (Week 8), the instructor (the fourth author) designed three components for classes during Week 2 to Week 7, including online lectures, group-level collaborative discussion, and collaborative writing of literature review (see Fig. 1). The first component, namely online lectures, covered main theories and concepts of the field and advanced content, i.e., advanced marine metastructures, artificial intelligence in engineering, and structural health monitoring in marine engineering. The second component was the group’s collaborative discussion, in which students explored the concepts, exchanged understandings, and constructed meanings in small groups through DingTalk. The instructor provided prompting questions to guide students’ inquiry of research topics in advance; and the instructor was not engaged in the discussion process. The third component was collaborative writing of literature review completed by the groups and the topic of the literature review was “Nanoscale Fabrication and Characterization of Mechanical Metamaterials”. The groups completed five collaborative writing tasks in a weekly base during Week 2 to Week 6 and finished the final literature review in Week 7 (see Appendix A, Table 1). In Week 8, student groups conducted online oral presentations to the class based on the literature review.

**Table 1:** Input and output variables

Variable	Type	Description	Symbol
Input	Prerequisite knowledge	A student’s prerequisite knowledge and weekly test scores	${\mathrm{PK}}_{\mathrm{s}}$
	Participation frequency	A student’s participation frequency in the group’s discussion	${\mathrm{Par}}_{\mathrm{group}}$
	Discussion depth	Evaluation of a student’s discussion content depth; the depth is scored as superficial-level knowledge (SK), medium-level knowledge (MK), and deep-level knowledge (DK); a final weighted score was calculated as 1N_SK_ + 2N_MK_ + 3N_DK_, where N represents the occurrence frequency of a code)	${\mathrm{Dep}}_{\mathrm{dis}}$
	Procedural performance	The instructor’s evaluation score of the group’s write-up	${\mathrm{Perf}}_{\mathrm{write}}$
Output	Learning effectiveness	A student’s weekly learning performance	${\mathrm{Lrn}}_{\mathrm{eff}}$

### Research intervention

**Fig. 2:**
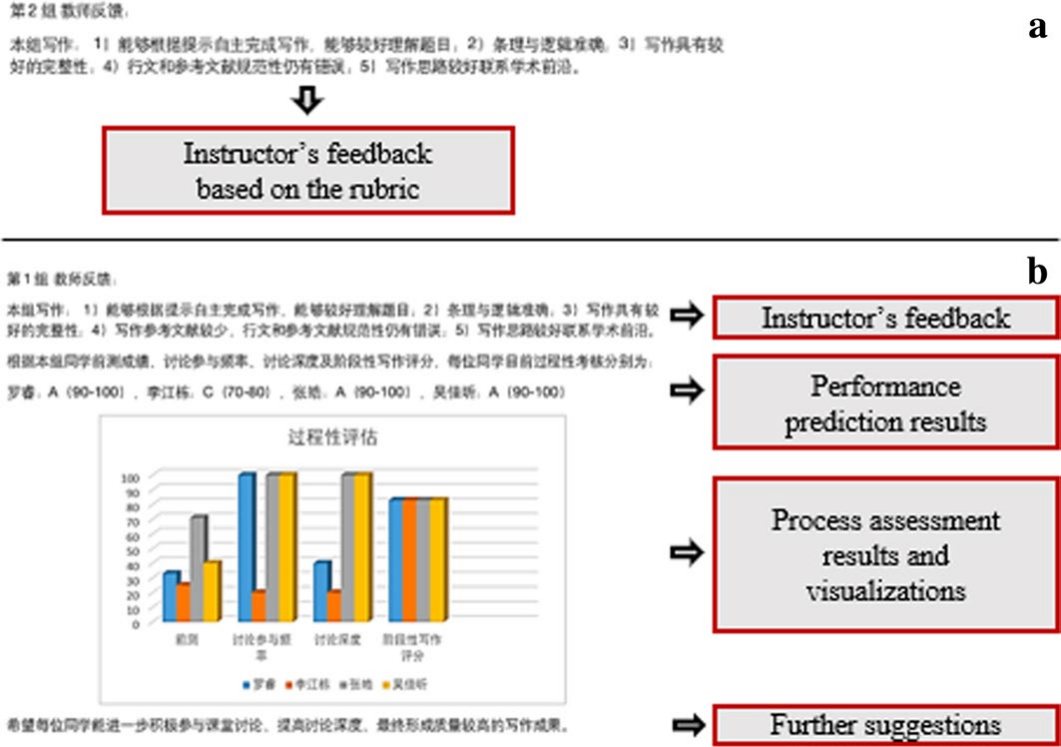
The integrated AI and LA approach

An integrated AI and LA approach was used as the research intervention. 15 groups were randomly assigned into the control condition (7 groups) and the experimental condition (8 groups). The control groups received the instructor’s feedback about the write-ups (see Fig. 2a). The experimental condition applied an AI model to predict students’ performances, and used LA approaches to visualize the results and provide feedback to the course instructor and students. Specifically, the experimental groups received the prediction performance of each student generated by the AI model, bar chart of the process assessment results, as well as further learning suggestions (see Fig. 2b). Students in a group can view their own performances as well as peers’ performances. The feedback was uploaded to each group’s DingTalk space in a weekly base after the group completed collaborative discussion and submitted the group’s write-up file.

Specifically, the instructor’s feedback about the write-up included five dimensions, namely understanding of the topic, related theory and concepts, technological challenges, logics and formats, as well as completeness (see Appendix A, Table 2). The AI-enabled academic performance prediction model was adopted from a previously proposed model developed by the same research team (Jiao et al., 2022). This model used genetic programming (GP) to accurately and efficiently predict the students’ academic performance based on students’ process-oriented data. The adopted model for the current research included four process-oriented input variables, namely the prerequisite knowledge (i.e., ${\mathrm{PK}}_{\mathrm{s}}$), the discussion participation frequency (i.e., ${\mathrm{Par}}_{\mathrm{group}}$), discussion depth (i.e., ${\mathrm{Dep}}_{\mathrm{dis}}$), as well as the procedural performance (i.e., write-up performance ${\mathrm{Perf}}_{\mathrm{write}}$). The output, namely learning effectiveness, was the learning performance of each student. The final prediction model was given as (Jiao et al., 2022):

**Table 2:** Content analysis framework of the discussion content

Code	Description	Example
Question elicitation (Qel)	A participant asked questions to elicit others’ perspectives or ideas	1.What is your understanding of this part? 2.Should the part of technical challenge focus on the application of metamaterials?
Information sharing (Ish)	A participant shared information that represented others’ perspectives from articles or resources without proposing his/her own perspective	1.Mechanical metamaterials are a class of man-made materials with special mechanical properties 2.A sharing of reference: Baek, D., Sang, H. L., Jun, B.H., & Lee, S.H. (2021). Lithography technology for micro- and nanofabrication
Perspective proposal (Ppr)	A participant proposed his/her own perspectives or ideas or responded to others without any explanations	1.Yes, it’s about the difficulty of using 3D printing to achieve special mechanical properties 2.I feel the same way and agree with you
Perspective elaboration (Pel)	A participant elaborated or responded perspectives or ideas supported with evidence, reasons, or argumentations	1.As photolithography is frequently used in applications, we should introduce several examples of applications, otherwise it is too general 2.Most of the marine engineering needs to explore the seabed, carry out excavation and other operations. Thus, we need to develop a variety of robots that can work underwater
Group regulation (GR)	A participant asked, understood or responded to the group task, made plans, monitored the group’s collaborative progress, and made reflections of the group progress	1.Please send the work to me by 10 p.m. today, and I'll sort it out and submit it 2.It’s my turn to integrate our work this week

1 $${\mathrm{Lrn}}_{\mathrm{eff}}=747.1-\frac{104200\mathrm{cos}\left({\mathrm{Perf}}_{\mathrm{write}}\right)}{{{\mathrm{Dep}}_{\mathrm{dis}}}^{3}}+{7.1\mathrm{cos}\left(\mathrm{cos}\left(\sqrt{{\mathrm{PK}}_{s}}\right)\right)}^{\mathrm{cos}\left(\mathrm{cos}\left({\mathrm{Perf}}_{\mathrm{write}}\right)\right)}+676.4\mathrm{cos}\left(\mathrm{cos}\left(\mathrm{log}\left({\mathrm{Par}}_{\mathrm{group}}\right)\right)\right)+0.7 {\mathrm{Par}}_{\mathrm{group}}$$

Four process-oriented input variables were calculated manually, which were then input into the prediction model and calculated in Matlab. The results were finally visualized in Excel and reported to the students by the research team.

### Data collection and analysis

**Fig. 3:**
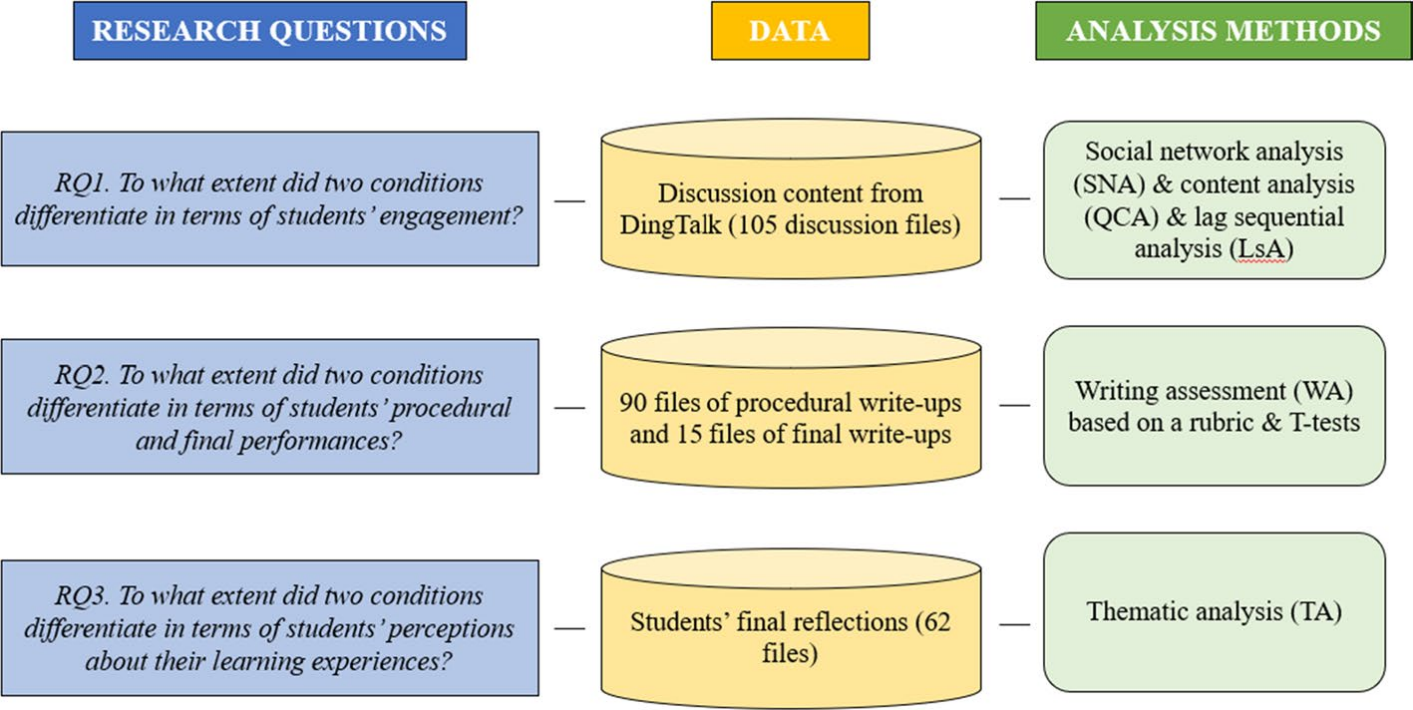
The analytical framework

The research data included three types. The first type was process data, namely the groups’ collaborative discussion content from DingTalk (105 discussion files in total, 15 groups * 7 weeks). The second type was performance data, namely the final write-up files of groups’ literature reviews (15 files), as well as 90 files of groups’ procedural write-up files (15 groups * 6 tasks). The third type was students’ perception data, namely the final personal reflections (62 files) (see Appendix B). A multi-method approach (including social network analysis, quantitative content analysis, and thematic analysis) was used in this research (see Fig. 3). Furthermore, based on the results, multiple t-tests were conducted to examine the differences between two conditions on student engagement, group performances, and learning perceptions.

This research used both quantitative and qualitative research methods to answer our research questions. To answer the first research question, this research focused on examining students’ social and cognitive engagement. This research used social network analysis (SNA) and quantitative content analysis (QCA) to analyze those two types of engagement dimensions. On the social engagement dimension, this research used the group-level SNA metrics to reveal groups’ social engagement attributes, including *density, average path length (APL), average degree, average closeness, average betweenness, global clustering coefficients (GCC), centralization, the inverse coefficient of variation (ICV) of interaction* (refer to Table 2 in Li et al., 2022). We first made network data in the excel files to record students’ interaction with peers at the group level. When A responded B with the symbol @, it was assigned as an interaction from starter A to receiver B. Since students sometimes did not refer to a specific peer during the online discussions, it was difficult to identify the receiver; in this situation, the receiver was denoted as “all”. For the *ICV* metric that needed to consider interactions between two identified students, we excluded the data of “all” in the SNA measure processes. In addition, SNA plots were drawn to visually demonstrate differences between two conditions. R packages *sna, igraph* and *tnet* were used to calculate SNA metrics and Gephi was used to visualize the social interaction network plots. T-tests were used to compare the differences of two conditions on the groups’ social engagement.

A framework was used to analyze students’ discussion content in DingTalk (see Table 2). Two research assistants coded the discussion content based on the coding scheme, reached a Cohen’s Kappa’s interrater reliability of 0.748 initially, then discussed and consulted with the first author to resolve conflicts, and reached an agreement of 100%. T-tests were also used to compare the differences of two conditions on the groups’ cognitive engagement and group regulation. Moreover, lag sequential analysis (LsA) approach was used to examine the differences of sequential patterns under two conditions. The R package *LagSeq* was used with the coded dataset.

To answer the second research question, this research made the groups’ procedural and final performances assessment based on a rubric. The rubric included five dimensions, namely understanding of the topic, related theory and concepts, technological challenges and solutions, formats, as well as completeness and logics (see Appendix A, Table 2). T-tests were used to compare the differences of two conditions on the groups’ procedural and final performances.

To answer the third research question, we used a thematic analysis method to analyze students’ final reflections, with inductive and deductive approaches (Nowell et al., 2017). Six steps were followed to identify themes and sub-themes: (1) formatting, reading, and becoming familiar with the reflection data, (2) coding the data independently by two research assistants in an inductive way, (3) resolving conflicts regarding codes through meetings between two research assistants, (4) comparing segments with same codes and collated codes into themes in a deductive way, (5) meeting with the first author to yield recurrent codes and themes by organizing the data around significant thematic units, and (6) reading the whole dataset and double-checking the final coded themes and sub-themes. Three themes and 11 sub-themes were derived.

## Results

### RQ1. To what extent did two conditions differentiate in terms of students’ engagement?

**Fig. 4:**
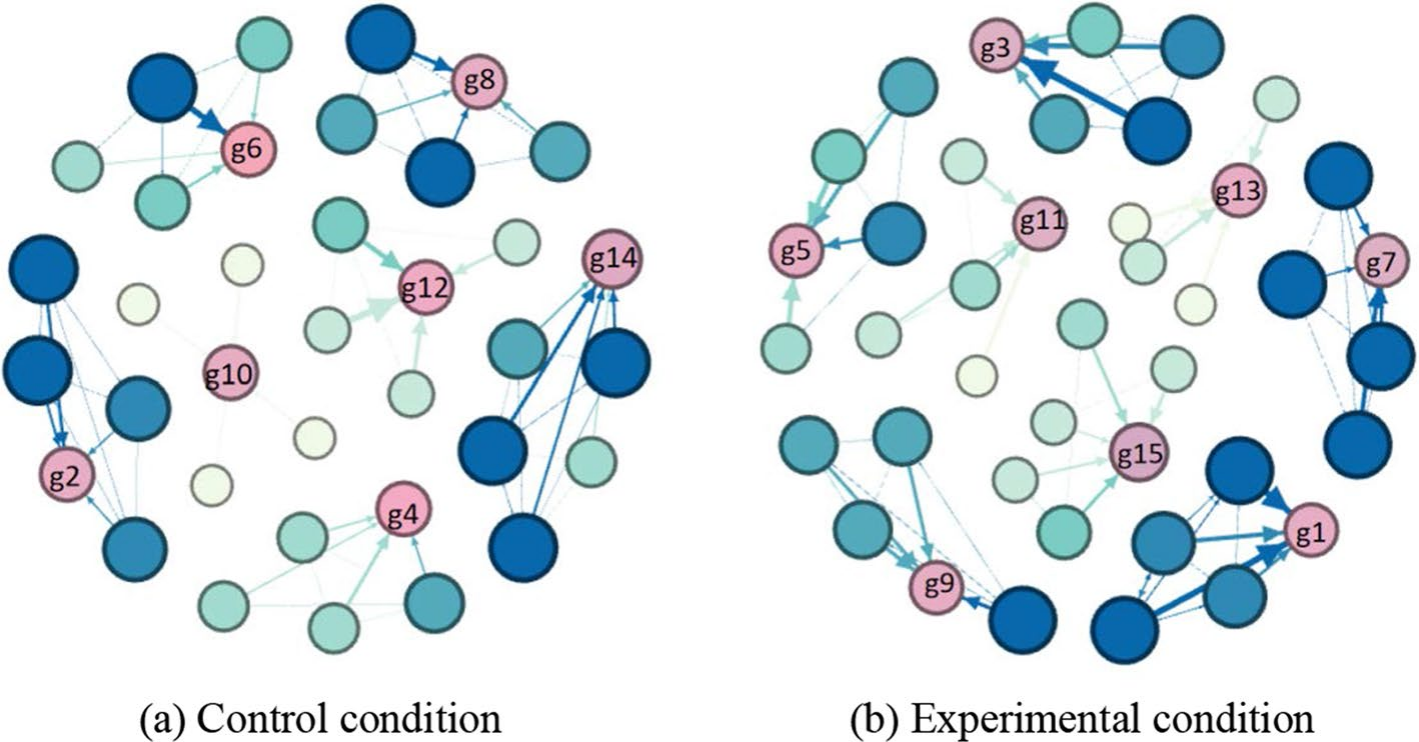
Social interaction network plots under two conditions. The node size represented interaction frequencies, the edge thickness represented the interaction frequencies that a student had to another student or the group; the node in blue represented one group member, the node in pink represented the whole group rather than one group member

First, regarding the social engagement, this research used T-tests to examine whether there were statistical differences in the SNA measurements between two conditions (see Table 3). The results showed that there were significant differences in most SNA measurements, including density (F = 4.90, p = 0.000), APL (F = 7.92, p = 0.039), average degree (F = 4.83, p = 0.000), average closeness (F = 1.80, p = 0.12), average betweenness (F = 10.39, p = 0.030), GCC (F = 1.80, p = 0.006) and centralization (F = 0.78, p = 0.007). On the abovementioned metrics, except the average closeness, the experimental groups had higher values of density, APL, average betweenness, GCC, and centralization than the control groups. In addition, although there was no statistical significance regarding ICV, the experimental groups had a higher ICV value than the control groups. The social network plots also showed that groups under the experimental condition had higher interaction frequencies than the groups under the control condition (see Fig. 4). Overall, the use of the integrated approach had a competitive advantage in maintaining more social engagement between members during the collaborative process.

**Table 3:** The comparison of social engagement between two conditions

SNA metrics	Condition	Mean	Std	F	*p*	Comparison
Density	Control	1.41	1.06	4.90	0.000***	Exp > Control
	Exp	2.44	1.39			
APL	Control	1.06	0.11	7.92	0.039*	Exp > Control
	Exp	1.11	0.14			
Average degree	Control	5.46	4.25	4.83	0.000***	Exp > Control
	Exp	9.52	5.64			
Average Closeness	Control	0.62	0.22	1.80	0.012*	Control > Exp
	Exp	0.50	0.24			
Average Betweenness	Control	0.14	0.29	10.39	0.030*	Exp > Control
	Exp	0.29	0.38			
GCC	Control	0.33	0.35	1.80	0.006**	Exp > Control
	Exp	0.51	0.33			
Centralization	Control	2.10	1.98	0.78	0.007**	Exp > Control
	Exp	3.29	2.42			
	Exp	0.05	0.07			
ICV	Control	1.86	0.94	0.03	0.535	–
	Exp	2.18	1.00			

* *p* ≤ 0.05; ** *p* ≤ 0.01; *** *p* ≤ 0.001

Second, this research used T-tests to examine whether there were statistical differences between two conditions on students’ cognitive engagement and group regulation (see Table 4). The results showed that there was no significant difference in most engagement codes, except Information sharing (Ish: F = 0.91, p = 0.003). The experimental groups had a higher contribution of Ish than the control groups. Though there were no statistical significances in other engagement codes, the experimental groups had the higher-level contributions on all engagement codes (i.e., Qel, Ppr, Pel, and GR) than the control groups. This result indicated that the experimental groups had a more active cognitive engagement and group regulation than the control groups.

**Table 4:** The t-test comparison of engagement codes between two conditions

Engagement codes	Condition	Mean	Std	F	*p*	Comparison
Question elicitation (Qel)	Control	0.35	0.80	3.29	0.187	
	Exp	0.57	0.91			
Information sharing (Ish)	Control	9.92	13.07	0.91	0.003**	Exp > Control
	Exp	18.52	15.58			
Perspective proposal (Ppr)	Control	7.78	11.20	1.40	0.244	
	Exp	11.32	18.38			
Perspective elaboration (Pel)	Control	0.69	1.64	2.10	0.403	
	Exp	1.05	2.58			
Group regulation (GR)	Control	6.73	6.87	3.00	0.068	
	Exp	10.11	11.04			

* *p* ≤ 0.05; ** *p* ≤ 0.01; *** *p* ≤ 0.001

**Fig. 5:**
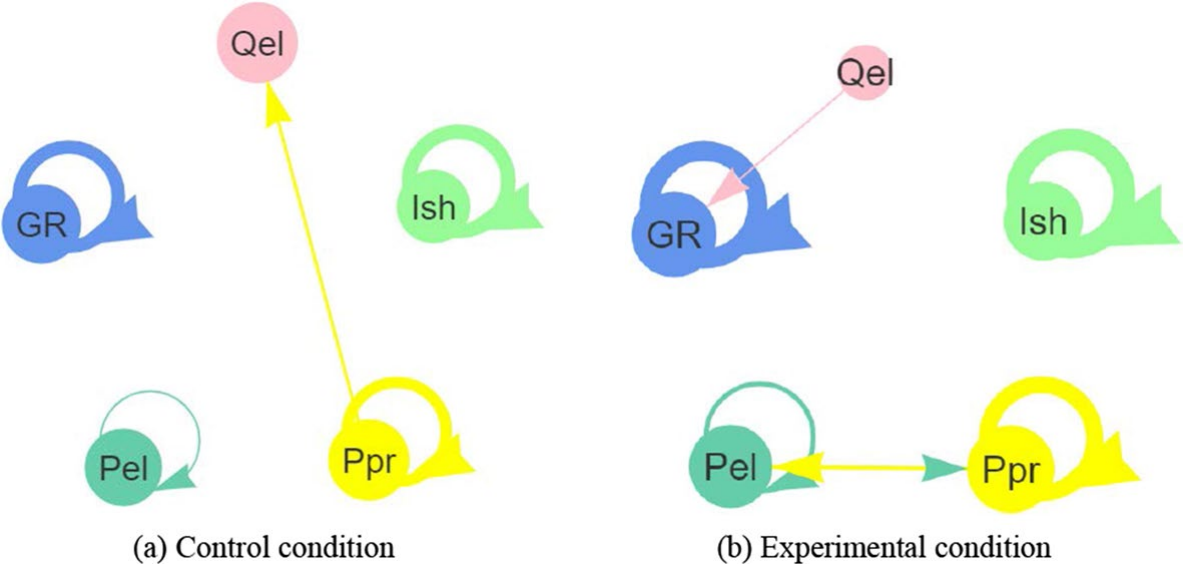
Transitions between two codes under two conditions

The LsA results showed that there were 5 types of transitions under the control condition with the Z score greater than 1.96 (see Table 5 and Fig. 5). The strongest transition was Ish—> Ish (Z score = 17.74), followed by GR—> GR (Z score = 16.45), Ppr—> Ppr (Z score = 13.83), Ppr—> Qel (Z score = 3.61), and Pel—> Pel (Z score = 2.21). There were 7 types of transitions under the experimental condition with a Z score greater than 1.96 (see Table 5 and Fig. 5). The strongest transition was Ish—> Ish (Z score = 24.2), followed by GR—> GR (Z score = 22.04), Ppr—> Ppr (Z score = 18.85), Pel—> Pel (Z score = 5.46), Ppr—> Pel (Z score = 4.72), Pel—> Ppr (Z score = 2.35), and Qel—> GR (Z score = 2.17). Compared to the control condition, the sequential transitions related to students’ perspectives (i.e., Ppr and Pel) were stronger under the experimental condition. Particularly, there were a bidirectional transition between Ppr and Pel, which indicated that students’ presentation of perspectives further promoted the elaboration of perspectives, and in turn facilitated further proposal of perspectives under the experimental condition.

**Table 5:** The adjusted residuals Z scores under two conditions

	Qel		Ish		Ppr		Pel		GR	
	Control	Exp	Control	Exp	Control	Exp	Control	Exp	Control	Exp
Qel	1.62	0.86	− 1.82	− 2.24	1.49	0.51	0.8	− 0.92	− 0.27	2.17*
Ish	− 2.81	− 2.6	17.74*	24.20*	− 7.97	− 12.02	− 1.86	− 4.05	− 9.88	− 13.38
Ppr	3.61*	1.31	− 9.89	− 12.7	13.83*	18.85*	1.36	4.72*	− 4.95	− 6.94
Pel	− 0.7	0.21	− 0.8	− 4.06	0.99	2.35*	2.21*	5.46*	− 0.78	0.21
GR	− 0.83	1.33	− 8.5	− 12.76	− 6.39	− 6.64	− 0.39	− 1.95	16.45*	22.04*

Z scores greater than 1.96 means that the transitional sequence reached a statistical significance, i.e., p < 0.05

### RQ2. To what extent did two conditions differentiate in terms of students’ procedural and final performances?

This research used T-tests to examine whether there were statistical differences in procedural and final group performance between two conditions (see Table 6). The results showed the experimental groups had the higher scores in most procedural and final performances except Task 4 where experimental groups had a slightly lover score than the control group. In addition, the experimental groups achieved significantly higher scores than the control groups in Task 2 (F = 17.65, p = 0.000), Task 5 (F = 9.07, p = 0.002), Task 6 (F = 3.06, p = 0.000), and the final write-up of literature review (F = 4.79, p = 0.008). Therefore, applying the integrated approach enabled the experimental groups outperformed the control groups in the procedural and final writing tasks.

**Table 6:** The t-test comparison of group performances between two conditions

Performance	Condition	Mean	Std	F	*p*	Comparison
Procedural performance (Task 1)	Control	78.90	1.93	5.02	0.496	–
	Exp	79.21	1.65			
Procedural performance (Task 2)	Control	76.21	1.80	17.65	0.000***	Exp > Control
	Exp	79.03	3.30			
Procedural performance (Task 3)	Control	72.14	8.64	3.68	0.925	–
	Exp	72.33	7.69			
Procedural performance (Task 4)	Control	75.59	6.39	16.22	0.489	–
	Exp	74.61	4.33			
Procedural performance (Task 5)	Control	81.62	1.74	9.07	0.002**	Exp > Control
	Exp	83.70	3.23			
Procedural performance (Task 6)	Control	81.07	1.93	3.06	0.000***	Exp > Control
	Exp	83.82	2.49			
Final performance (Literature review)	Control	87.97	1.21	4.79	0.008**	Exp > Control
	Exp	89.33	1.41			

* *p* ≤ 0.05; ** *p* ≤ 0.01; *** *p* ≤ 0.001

### RQ3. To what extent did two conditions differentiate in terms of students’ perceptions about their learning experiences?

There were three themes emerged in the thematic analysis of students’ reflection data, namely *perceptions of the feedback*, *effects of feedback on group’s collaborative learning,* and *attitudes toward online collaborative writing* (see Table 7).

**Table 7:** Themes and sub-themes extracted from student reflections under two conditions

Themes and sub-themes	Exp (N = 33)	Control (N = 29)
1. Perceptions of the feedback		
a) Timely offering and check of the feedback	33	25
b) Positive attitudes towards the feedback	28	20
c) Asking for more specific suggestions on writing	12	19
2. Effects of feedback on group’s collaborative learning		
a) Self-reflection about the group collaboration	18	3
b) Increased communications with peers	11	4
c) Regulations of collaborative writing	4	7
d) Improvement on the writing products	8	2
3. Attitudes toward online collaborative writing		
a) Sharing of information and expansion of ideas	15	11
b) Convenience to record, share, and regulate writing	13	10
c) No limitations of distance, location, or time	6	0
d) Inefficiency of collaborative writing or communication	9	12

The first theme revealed the students' *perceptions of the feedback*, including three sub-themes, namely *timely offering and check of the feedback, positive attitudes towards the feedback,* and *asking for more specific suggestions on writing*. All of the students (N = 33) in the experimental groups appreciated the timely feedback offered by the instructor and research team and reported that they checked the feedback on time. Among all students in the experimental groups, 28 students expressed a positive attitude about the feedback. For example, Wang mentioned, “*I focused more on the items that scored low and improved them in the next assignment. I also paid attention to the scores of other members in our group and ask other members for advice*.” In addition, 12 students in the experimental groups suggested that the instructor should provide more specific suggestions on how to improve the essay writing. For example, Zhang said, “*I hope the instructor could give more detailed suggestions, for example shortcomings of our logic, expressions or citation formats.*” In the control groups, 25 out of 29 students mentioned that they checked the feedback provided by the instructor. Comparing to the experimental groups, there were less students (N = 20) expressed a positive attitude towards the instructor’s feedback, while there were more students (N = 19) pointed out the necessity of concrete suggestions on essay writing. For example, Wu said, “*The feedback would be given in a more specifical way, such as problems we made in a particular paragraph.*” Overall, students in the experimental groups had more positive perceptions of the feedback provided and the instructor than the control groups.

The second theme revealed the *effects of feedback on group’s collaborative learning*, including four sub-themes of *self-reflection about the group collaboration, increased communications with peers, regulations of collaborative writing, and improvement on the writing products*. 18 out of 33 students in the experimental groups mentioned they made self-reflections about the group’s collaborative writing process, while only 3 student in the control groups mentioned this sub-theme. For example, Zhang (the experimental group) reported “*I scored at the range of 80–90 in the first task… I reflected on it and found a lack of depth of discussion in my writing.*” And Wang from the control groups said “*I would pay more attention to the instructor’s feedback in my next assignment*.” 11 students in the experimental groups reported that they had more communications and interactions with their peers, while only four students in the control groups mentioned this sub-theme. For example, Wang (the experimental group) reported “*When I found the frequency and depth of my discussion was low in the feedback chart, I would make efforts to engage in the discussion the next time.*” And Tao (the control group) said “*The feedback helped us improve our group interaction at the beginning of the course.*” Moreover, 8 students in the experimental groups reported an improvement on the group’s writing products, as Li said “*We improved the quality and format of the content based on the instructor’s feedback.*” In contrast, only 2 students in the control groups referred to the positive effects of the instructor’s feedback to the final essay. Finally, four students in the experimental groups mentioned the regulation of collaborative writing while seven students in the control group mentioned this sub-theme. Overall, the feedback provided by the research team and the instructor had more positive effects on the experimental groups than the control groups.

The third theme revealed the students' *attitudes toward online collaborative writing*, including four sub-themes, namely *sharing of information and expansion of ideas, convenience to record, share, and regulate writing, no limitations of distance, location, or time, and inefficiency of collaborative writing or communication*. Students in both the experimental groups (N = 15) and the control groups (N = 11) mentioned that online collaborative writing made it convenient to share information and discuss and integrate ideas. They mentioned that the collaboration made it possible to record, share, and regulate writing and reconciliate the group's division of tasks (The experimental groups: N = 13; the control groups: N = 10). For example, Wu (the experimental group) reported that, “*My teammates were responsible…It was straightforward and easy to share documents and links in the collaboration.*” Zhang (the control group) said, “*We had a clear division of labor, and it is easy to record and share information by screenshots, video recordings and links.*” In addition, 6 out of 33 students in the experimental groups thought there was no limitation of distance, location, or time in online collaborative writing, while no students in the control groups mentioned this advantage of online collaborative work. Moreover, it was worth noting that both students in the experimental and control groups had encountered inefficiencies of collaborative writing and group communication, compared to the face-to-face learning environment (The experimental groups: N = 9; control groups: N = 12). Overall, students in the experimental groups and control group held similar attitudes towards the online collaborative writing activities.

## Discussions

### Addressing research questions

This research presented an integrated approach of AI performance prediction model and learning analytics approaches to address some of the main concerns associated with the application of AI in education. To improve the actual teaching and learning effects, this research applied an AI model in an online engineering course to predict students’ performances, used LA approaches to visualize the results and provide feedback to the course instructor and students, and further conducted a quasi-experimental research to compare the effects of student learning with and without the support of the integrated approach. The results showed that the experimental groups outperformed the control groups in terms of the group students’ engagement, procedural and final performances, as well as students’ perceptions of learning experiences. Regarding the first research question, the result showed that, compared to the control condition without the integrated approach support, the use of AI and LA approaches had a competitive advantage in maintaining students’ social and cognitive engagement during the collaborative learning process. Specifically, students under the experimental groups had more social interactions with peers and more transitional communications of perspective proposal and elaboration. Regarding the second research question, the result showed that applying the AI prediction model enabled the experimental groups outperformed the control groups in the procedural collaborative writing tasks as well as the final writing task. Regarding the third research question, the result showed that the experimental groups had more positive attitudes and perceptions of the feedback provided by the integrated approach and the instructor than the control groups, including more self-reflections about writing, more peer communications, and better improvement of the writing products, and less chances for asking for extra feedback and assistance and less feelings about inefficiency of the collaborative writing. Overall, the application of AI prediction model and LA visualization and feedback had competitive advantages to increase collaborative engagement, improve the group collaboration performances, and improve student satisfaction about the collaborative learning activities.

### Implications for the implementation of AI-enabled academic performance prediction model

AI performance prediction has become an emerging direction to analyze complex data in education by AI algorithms and predict learning performance of students with satisfactory accuracy. Comparing with its counterparts in the traditional statistics, AI takes advantage of its computation superiority to achieve the analysis, and therefore, results that are otherwise unfinishable in traditional classes. Studies have been conducted to predict learning performance using nearly all types of AI algorithms, such as the algorithms in the major areas of ML, EC, nature language processing (NLP), computer vision (CV), etc. (Ouyang & Jiao, 2021). However, lack of research attention has been paid on the next step, i.e., using the AI prediction results to improve teaching and learning effect. To address these challenges, one direction is to further improve the computation power of AI prediction models by combining different algorithms, and another solution is to develop the learning effectiveness criteria using well-established approaches in other fields such as analytic hierarchy process (AHP). On the one hand, comprehensive AI prediction models require hybrid algorithms as they are too complex to be analyzed by any individual AI algorithm. For example, robotics in AIEd have been reported with the integration of ML, planning, and sometimes KRR with hardware systems; cognitive modeling has been developed to build hybrid systems of human thinking and behavior such as the ACT-R system; and knowledge representation and reasoning have been applied in various question-answering systems (Tahiru, 2021). On the other hand, comprehensive AI prediction models require well-defined criteria to evaluate the effectiveness. For example, AHP can be used to build an evaluation system that takes into account the input factors and their consideration weights, and therefore, establish the feedback and early-warning-intervention function to complete the closed loop of AIEd (Drake, 1998). This research has moved a step forward to closing the loop between developing AI performance prediction models *from the AI model perspective* to applying AI prediction models and examining the model’s effects *from the educational application perspective.* Particularly, this research proposed an integrated approach of AI models and LA approaches to close this loop. However, the current work required the researchers to process the student characteristics data and manually used the data and AI algorithm model to generate feedback. Future work under the educational application strand can further design automatic data collection and analytics functions and develop AI prediction evaluation system to provide in-time feedback in order to improve the quality of teaching and learning.

### Implications for AI-driven learning analytics and educational data mining

AIEd transforms the instructor and student roles and agencies, offers individualized, personalized, student-centered learning experiences, and fosters knowledge connections, creation and knowledge network building (Ouyang & Jiao, 2021). The learning sciences community has called for the integration of AI methods in learning analytics and educational data mining, which can better deal with complex, nonlinear information, extract and represent multi-level and high-dimensional features of student learning, compared to traditional learning analytics, such as social network analysis or content analysis (de Carvalho & Zárate, 2020). In addition, the integration of AI and LA can support instructors’ informed decision making to facilitate student-centered learning and improve student groups’ knowledge construction processes. AI performance prediction model, as a type of AI-driven tool, can foster students’ awareness and self-reflection of the learning process with an expectation that purposeful use of the tool can boost student engagement and further improve their learning quality. However, most previous AI prediction models mainly focus on the evaluation of students’ summative performance rather than the process-oriented analytics, which may result in a lack of a comprehensive, holistic understanding and intervention of the learning process. The current work integrated AI and LA approaches with both performance and process data to reveal the characteristics of student groups’ collaborative learning. Future work can use advanced AI algorithms (e.g., natural language processing, genetic programming) with learning analytics and data mining to offer in-time, multidimensional characteristics of collaborative learning (Amon et al., 2019; de Carvalho & Zárate, 2020; Hoppe et al., 2021). Overall, AI-driven learning analytics and educational data mining has potential to provide feedback on similarities and differences of student performance at the individual and group level to close the loop of AI model development and educational application, with an ultimate goal of improving the instruction and learning quality.

### Paradigmatic implications for the closed loop of AI development and educational application

To ensure a successful paradigm shift in the future, AIEd development should close a loop of AI model development and educational application. The loop includes four steps, namely a) AI model development, b) model and algorithm optimization, c) AI model educational application, and d) validation through empirical research (Wu et al., 2022; Xie et al., 2019; Yang et al., 2021). Within this loop, the incorporation of human domain knowledge and experience is beneficial for promoting the automation of AI models. Particularly, the real-time AI algorithm model and visualized LA feedback and guidance is an essential component to ensure the multidimensional attributes and information demonstrated to support students’ learning. Many factors are critical in the educational context such as the cognitive, emotional, ethic dimensions, philosophical, social considerations. While admitting the fact that AI performs better computing and logic decision-making than human, it is extensively accepted that certain characteristics of human beings are unreplaceable, e.g., cognitions, emotions, feelings, perceptions, etc. (Yang et al., 2021). As a consequence, it is particularly important to approach AIEd from the human perspective by considering multidimensional attributes, conditions and contexts of students, instead of focusing on the AI algorithms and computation skills. The integrated approach of AI and LA abovementioned can assist this transformation. The closed loop of AIEd represents the integration of human intelligence and machine intelligence, where students should be centralized as the main focus and instructor should foster student-centered learning and decision making. As a previous work conceptualizes, three AIEd paradigms are emerged moving from the AI-directed, learner-as-recipient paradigm to the AI-supported, learner-as-collaborator paradigm, and finally towards the AI-empowered, learner-as-leader paradigm (Ouyang & Jiao, 2021). This research moves one step towards the paradigmatic shift by using the GP-based performance prediction model with learning analytics visualization and feedback. In addition, the empirical research combined the multimodal data analysis (including process, performance, and perception), which provides is an effective way to examine the effect of the integrated approach on learning. Overall, this research gives researchers and practitioners an integrated approach to incorporating advances in AI-driven learning analytics by having a definition of educational loop and grounding in a paradigmatic model of relevant education or learning processes.

## Conclusions

Future teaching and learning should focus on the integration of AI for learning analytics, with a goal to use of AI to organize, analyze, and understand data for decision-making and for supporting student success (Pelletier et al., 2022). Among various AI applications, AI performance prediction model is used to identify at-risk students, establish student-centered learning pathways, and optimize instructional design and development. A majority of the existing AI prediction models focus on the development and optimization of AI models and algorithms rather than applying AI models to provide in-time and continuous feedback and improve the student learning quality. To fill this gap, this research integrated an AI performance prediction model with learning analytics visualization and feedback and conducted quasi-experimental research in an online engineering course to examine the differences of students’ learning effect with and without the support of the integrated approach. Empirical research results showed competitive advantages of the application of this integrated approach on students’ engagement, performances and learning perceptions and experiences. Future work should expand the educational contexts, course subjects, as well as sample size to test and verify the empirical research results and implications. Overall, the contributions of this research centers on the following three aspects: (1) proposing an integrated approach to combine AI performance prediction results and LA feedback to foster student learning; (2) conducting empirical studies to investigate the effect of this integrated approach; and (3) providing implications to further develop AI-driven learning analytics and close the loop between AI model development and educational application.

## Data Availability

The data and materials that support the findings of this study are available on request from the corresponding author.

## Appendix A

See Tables 8, 9.

**Table 8:** The writing tasks and topics

Task	Topic	Outline
Literature review (The final write-up)	Nanoscale Fabrication and Characterization of Mechanical Metamaterials	1.Introduction 2.What is known in the literature 3.What is unknown/limitation 4.The aim of this technical review 5.Review outline 6.Conclusions
Task 1 (The procedural write-up)	Micro/Nanoscale Fabrication of Mechanical Metamaterials Based on 3DPrinting	1.Advent and Development of Micro/Nanoscale 3DPrinting 2.Main Applications of Micro/Nanoscale 3D Printing 3.Micro/Nanoscale 3D Printing in Mechanical Metamaterials 4.Technological Challenges 5.Conclusions
Task 2 (The procedural write-up)	Micro/Nanoscale Fabrication of Mechanical Metamaterials Based on Photolithography	1.Advent and Development of Micro/Nanoscale Photolithography 2.Main Applications of Micro/Nanoscale Photolithography 3.Micro/Nanoscale Photolithography in Mechanical Metamaterials 4.Technological Challenges (Name of the 4th group member/Writer) 5.Conclusions
Task 3 (The procedural write-up)	Micro/Nanoscale Fabrication of Mechanical Metamaterials Based on [TECHNOLOGY 1] and [TECHNOLOGY 2]	1.Development and Main Applications of [TECHNOLOGY 1] 2.[TECHNOLOGY 1] in Fabrication of Mechanical Metamaterials 3.Development and Main Applications of [TECHNOLOGY 2] 4.[TECHNOLOGY 2] in Fabrication of Mechanical Metamaterials 5.Conclusions
Task 4 (The procedural write-up)	Mechanical Testing and Characterization of Mechanical Metamaterials: Experimental Setup and Mechanical Testing	1.Experimental Devices for Micro/Nanoscale Mechanical Testing 2.Main Challenges in Micro/Nanoscale Mechanical Testing 3.Experimental Setup to Test Young’s Modulus and Poisson’s Ratio 4.Typical Procedures to Test Young’s Modulus and Poisson’s Ratio 5.5.Conclusions
Task 5 (The procedural write-up)	Mechanical Testing and Characterization of Mechanical Metamaterials: Mechanical Characteristics of Mechanical Metamaterials	1.Ultra-Stiffness and Ultra-Lightness 2.Negative Poisson’s Ratio and Other Negative Response 3.Self-Adaptive Response 4.Mechanic-Interdisciplinary Response 5. Conclusions

**Table 9:** Assessment rubric of the group’s write-up

	Below expectation (5 point)	Meet expectation (10 points)	Exceed expectation (15 points)
Understanding of the topic	Research problems or understandings of the topic are not clearly stated. An explanation is either not given or a weak connection is made as to why these problems or topics are relevant to the field of the research	Research problems or understandings of the topic are clearly stated. An explanation is given as to why these problems or topics are relevant to the field of the research	Research problems or understandings of the topic are clearly stated and briefly explained. A thoughtful and convincing explanation is given as to why these problems or topics are important and relevant to the research
Related theory and concepts	The theory or related concepts are not properly used. An explanation is not given as why the theory or concepts can be applied	The theory or related concepts are properly used. Explanation is given as why the theory or concepts can be applied in the research	The theory or related concepts are properly used and clearly stated. A convincing explanation is given as why the research is based on the theory or concepts
Technological challenges and solutions	Technological challenges and potential solutions are not clearly stated or some important part of technologies is missing	Technological challenges and potential solutions are briefly stated and appropriate for the research context	Technological challenges and potential solutions are clearly stated and skillfully applied and integrated into the research context
Formats	APA reference format is not fully followed or some reference lists are missing	APA reference format is fully followed	APA reference and in-text citation format are both correctly followed
Completeness and logics	The literature review represents a series if disjointed ideas that are not organized into a cohesive narrative	The literature review is organized in a smooth and cohesive structure	The literature review is a cohesive, synthesized work with a clear logic of presentation

## Appendix B

### Final reflection questions

1. We used online collaborative discussion and online collaborative writing in this course. Please recall your group’s collaboration process. How did you collaborate as a group? How did you assign collaborative work as a group? How did you address any conflicts arising from disagreements between group members?
2. What aspects do you like about the collaborative discussions in the group before writing? Do you think there are disadvantages of this instructional strategy?
3. After the collaborative writing, the instructor provided the following feedback questions to the groups:
  - Did you review the feedback? If no, why?
  - If you thought the feedback was not useful, why? Please give details explanations of why it was not useful for you. And what the instructor could do to make the feedback more effective? How?
  - If you thought the feedback was useful, what aspects did you like about it? How did it positively influence your learning?
4. Do you suggest the instructor to use similar feedback in the future? Do you have any further suggestions, e.g., what information or functions you would like to see in the future.
5. Please make some suggestions about how to better use this collaborative learning strategy in future instructional practices.
